# Supplementary material for: Intracellular sodium elevation reprograms cardiac metabolism
Source: Nat Commun. 2020 Aug 28;11:4337. doi: 10.1038/s41467-020-18160-x (PMC7455741; doi:10.1038/s41467-020-18160-x)
Supplement: Supplementary file 1 — Supplementary Information [file 41467_2020_18160_MOESM1_ESM.pdf]

Intracellular sodium elevation reprograms cardiac metabolism

Aksentijevic et al.

Supplementary Note 1

Cellular compartmentation of the ^{23}Na TQF NMR signal

Supplementary Figure1 Panel A shows a water-fall plot of a single experiment ^{23}Na TQF spectra and panel B shows the data averaged from 5 separate hearts for the saponin washout experiment. In Panel B, the open circle symbol represents the fraction of the TQF signal that is attributable to the intracellular compartment estimated in previous TmDOTP expriments.[1]

When the heart is exposed to a Na-free solution the washout of the vascular and extracellular space is very rapid (we have previously estimated this to be <40 seconds in isolated hearts using flame photometry). After this, there is a steady fall in the TQF signal as Na leaks out of the cell and intracellular Na falls. Previous studies using ion-selective microelectrodes[2] have reported that intracellular Na falls with a very rapid initial component. For example, in the experiments of Ellis,[2] in what are likely to be relatively poorly superfused Purkinje fibres, changing extracellular Na from 140 to 14mM causes a rapid fall in intracellular Na from 10 to 2.9mM with 90% of this change complete in 3.2 mins. This is very similar to the time course of intracellular Na decline we see in our experiments when switching to a Na-free solution.

1. On changing to the Na-free saponin-containing solution, the TQF signal continues to fall as the membrane is permeabilised. We were slightly surprised that saponin did not accelerate significantly the rate of fall of intracellular Na - suggesting that the rate of efflux before saponin was not significantly different to that after saponin. However, the TQF signal then fell to a plateau at about 9% of the baseline.
2. On exposure to Triton X-100, the remaining TQF signal was eliminated – presumably as the mitochondria were permeabilised.

On the basis of the data shown above, we therefore estimate the following compartmental contributions to the total TQF signal –

Vascular + extracellular space = 47%
Intracellular signal = 53%
Cytoplasmic signal = 44%
Mitochondrial signal = 9%

In the experiments reported in our study, we assume that the total Na in the vascular and extracellular compartments does not change acutely and simply provides an offset to the intracellular signal. In order to control for this eventuality, however, we measure the DQF signal. As we have previously shown,[1] the DQF signal entirely originates from the vascular and extracellular bound Na located in anisotropic environment. So, we use this DQF signal as an internal control to verify that this compartment is not changing and any changes in TQF are thus attributable to changes in the intracellular

compartment. It is interesting to note that while the mitochondria occupy about 40% of the cell volume, and have a similar internal Na concentration to that in the cytosol, they contribute only 20% of the intracellular TQF signal suggesting that Na electrostatic buffering in the mitochondria differs from that in the cytoplasm.

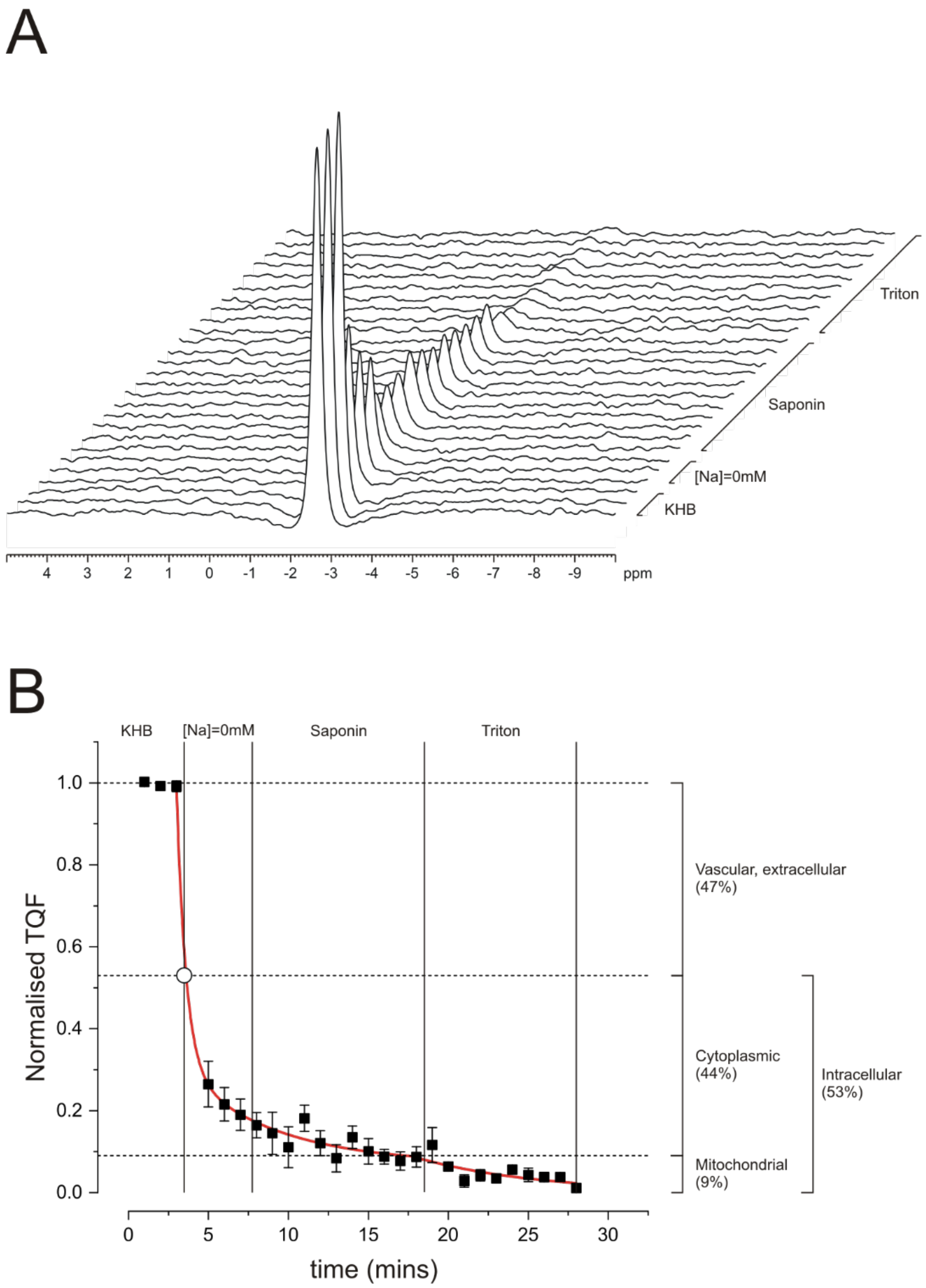

Supplementary Figure 1: **Triple Quantum Filtered ^{23}Na NMR spectroscopy assessment of the Intracellular Na compartmentation in perfused heart**
A) Water-fall plot of a time-series of single ^{23}Na TQF spectra during the saponin washout experiment. B) Average normalised ^{23}Na TQF intensity from n=5 separate hearts (biologically independent samples). The different washout treatment are labelled at the top of the time-course. The open circle symbol represents the fraction of the TQF signal that is attributable to the intracellular compartment estimated in our previous TmDOTP expriments.[1] Data are mean±SEM. Source data are provided as a Source Data file.

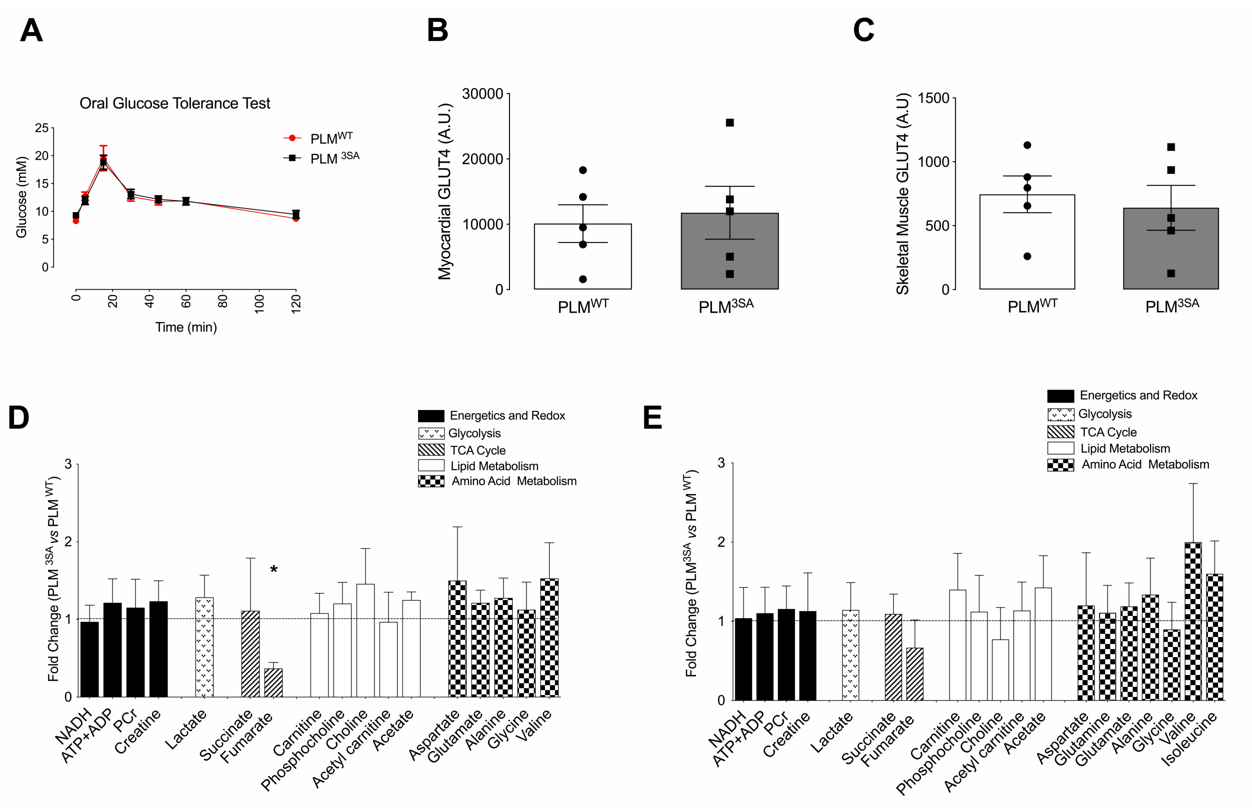

Supplementary Figure 2: Systemic metabolic phenotype of PLM^{3SA} mouse A) Glucose tolerance test results n = 8 biologically independent samples. B) GLUT4 expression in heart and C) skeletal muscle. n = 5 biologically independent samples. D-E) ^{1}H NMR metabolomic profiles - metabolite fold change normalized to control concentration (PLM^{WT} = 1) with propagated error (SEM) D) fed and E) post-overnight fasting skeletal muscles (gastrocnemius+soleus) (n=5 biologically independent samples). Data are mean ± SEM *P<0.01 vs PLM^{WT} control by Student t-test (two-tailed). Source data are provided as a Source Data file.

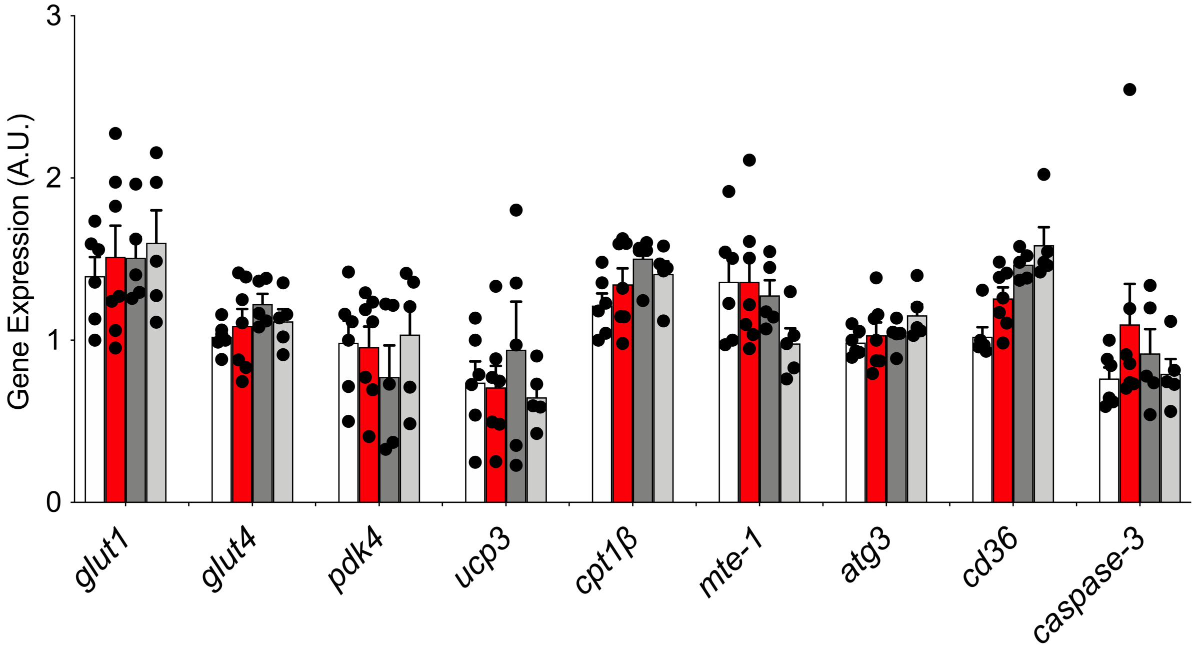

Supplementary Figure 3: Cardiac expression of genes involved in the regulation of mitochondrial metabolism. Mitochondrial thioesterase-1 (*mte-1*), pyruvate dehydrogenase kinase 4 (*pdk4*), uncoupling protein 3 (*ucp 3*), glucose transporters (*glut1*, *glut4*), fatty acid transporter (*cd36*), mitochondrial fatty acid transporter carnitine palmitoyltransferase 1 (*cpt1*), markers of apoptosis (*caspase-3*) and autophagy (*atg3*). Relative-quantity mRNA expression. Data are mean ± SEM (5 biologically independent samples). P>0.05 Comparisons by one-way ANOVA were subject to Bonferroni multiple comparisons post-test. Source data are provided as a Source Data file.

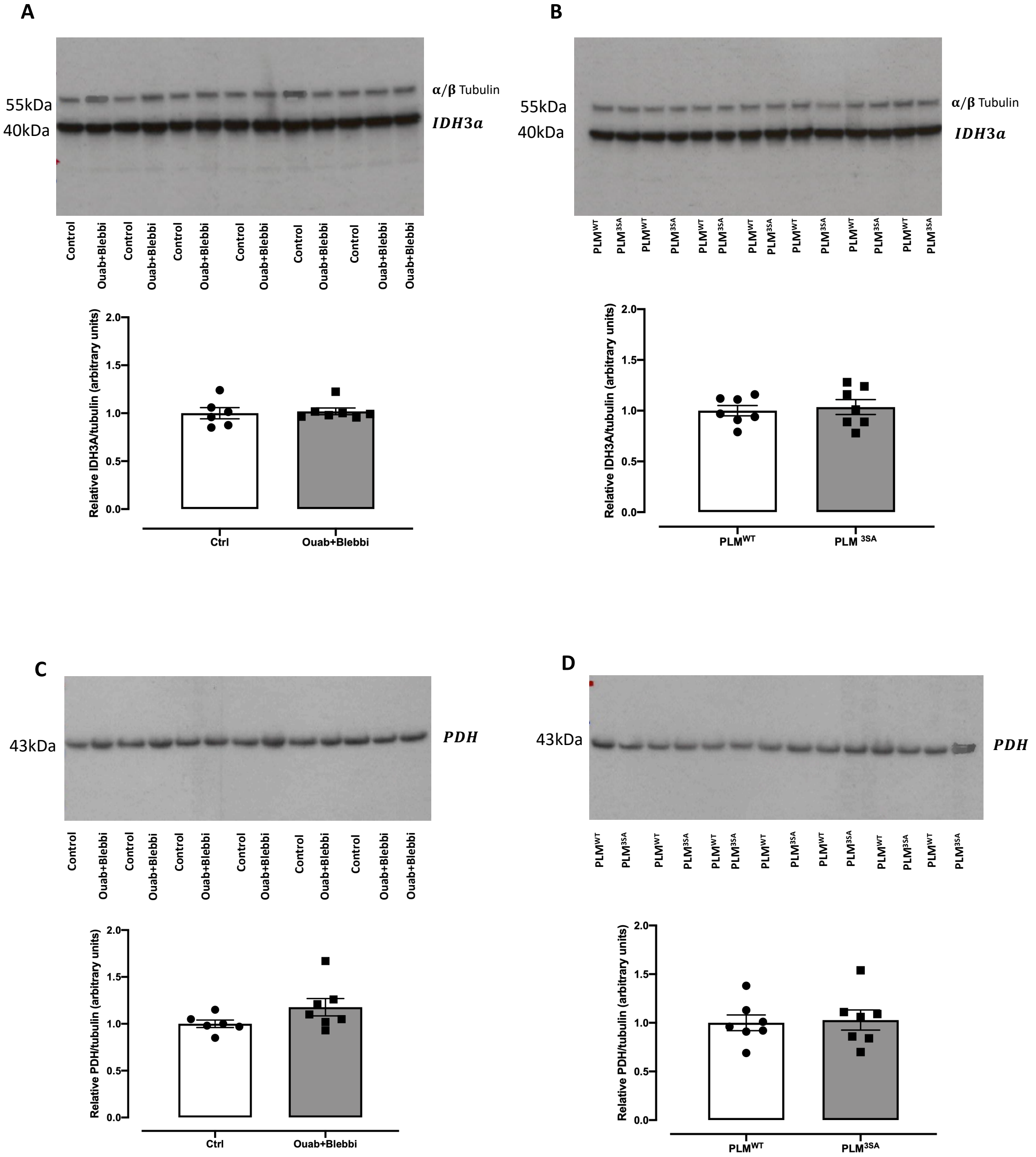

Supplementary Figure 4: Isocitrate dehydrogenase and pyruvate dehydrogenase expression in perfused hearts. Isocitrate dehydrogenase isoform 3α expression in A) acute sodium elevation ouabain+blebbistatin perfused hearts B) chronic sodium elevation PLM3^{SA} vs control PLM^{WT}. Pyruvate dehydrogenase (E2/E3) expression in C) acute sodium elevation ouabain+blebbistatin perfused hearts and D) chronic sodium elevation PLM3^{SA} vs control PLM^{WT}. Expression was normalized to anti-tubulin loading control. Data±SEM. n=8 control, n=7 ouabain+ blebbistatin treated; n=7 biologically independent samples. PLM^{WT}, PLM^{3SA} Comparison by Student t-test (two-tailed). Source data are provided as a Source Data file.

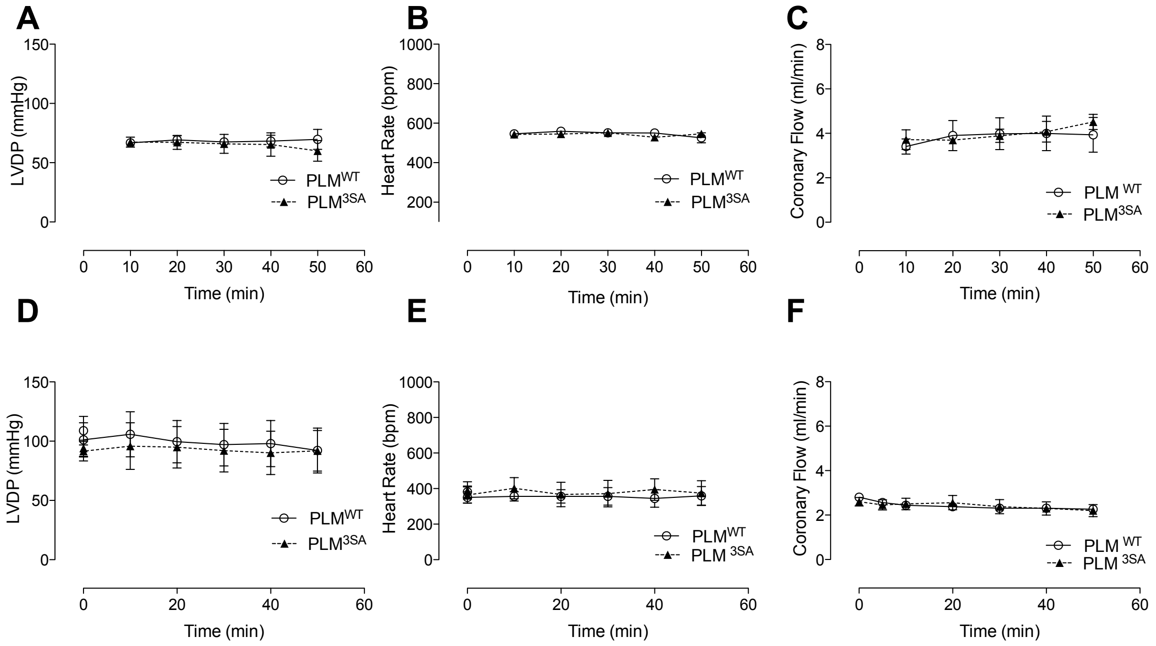

Supplementary Figure 5: Ex-vivo function of PLM^{WT} and PLM^{3SA} hearts. A-C) Left ventricular developed pressure (LVDP), heart rate and coronary flow in PLM^{WT} and PLM^{3SA} hearts perfused with crystalloid KH buffer paced at 550bpm with epicardial silver wire electrodes placed at the apex of the left ventricle and the right atrium. n = 6 biologically independent samples. D-F) Left ventricular developed pressure (LVDP), heart rate and coronary flow in unpaced PLM^{WT} and PLM^{3SA} hearts at intrinsic heart rate perfused with crystalloid KH buffer (n =6 biologically independent samples). Data presented as mean ± SEM. Source data are provided as a Source Data file.

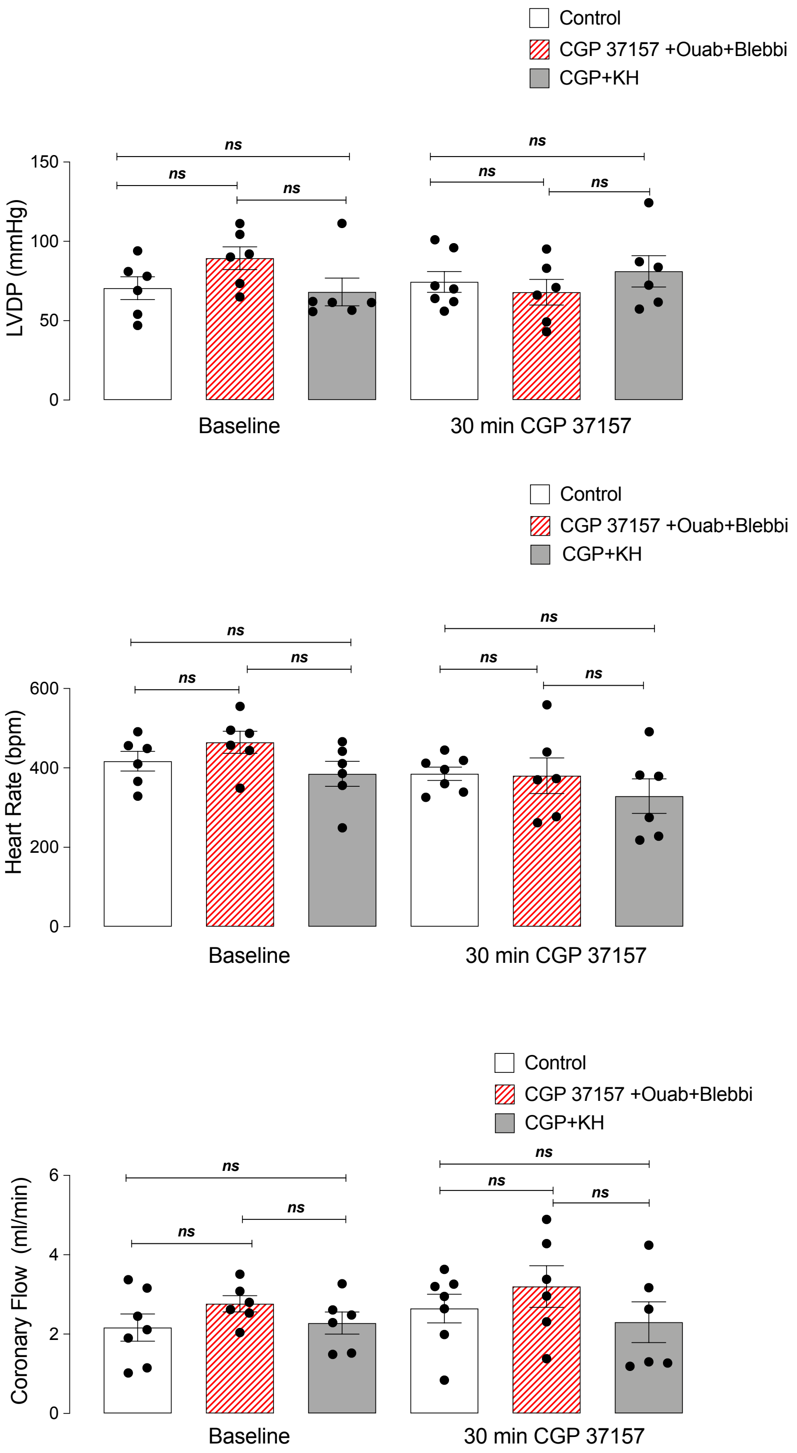

Supplementary Figure 6: Impact of the mitochondrial Na/Ca exchange inhibition with CGP37157 on cardiac function of acutely Na_i overloaded hearts. Impact of 30 min perfusion with 1 μmol/l CGP37157 in KH buffer on *ex vivo* function of C57/BL6J hearts and Ouab + Blebbi treated hearts. Baseline = equilibration, Treatment = end of 30min CGP37157 treatment. n=6 biologically independent samples. Data presented as mean ± SEM. Comparisons by one-way ANOVA were subject to Bonferroni multiple comparisons post-test. Source data are provided as a Source Data file.

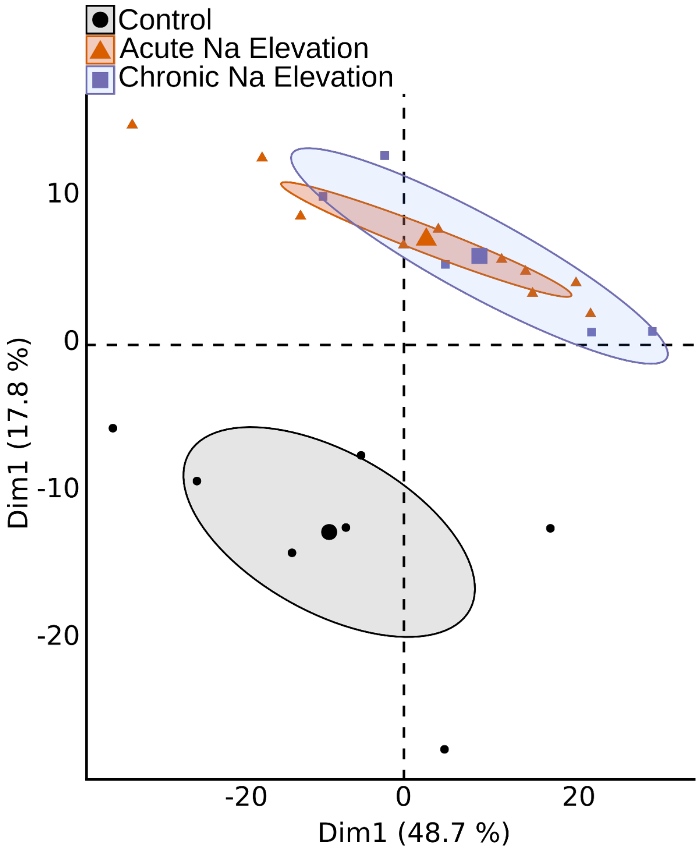

Supplementary Figure 7: Principal component analysis (PCA) of CardioNet simulation for control, acute and chronic Na_i elevation.

Ellipses indicate 95% confidence interval. Variables are centred around the mean of each group. The first two components of the PCA explain 71.4% of the variability. Sample segregated into control and Na_i elevation. Data represent simulations for each biological replicate in control (n = 7), acute Na elevation (n = 10), and chronic Na elevation (n = 5).Samples were biologically independent. Source data are provided as a Source Data file.

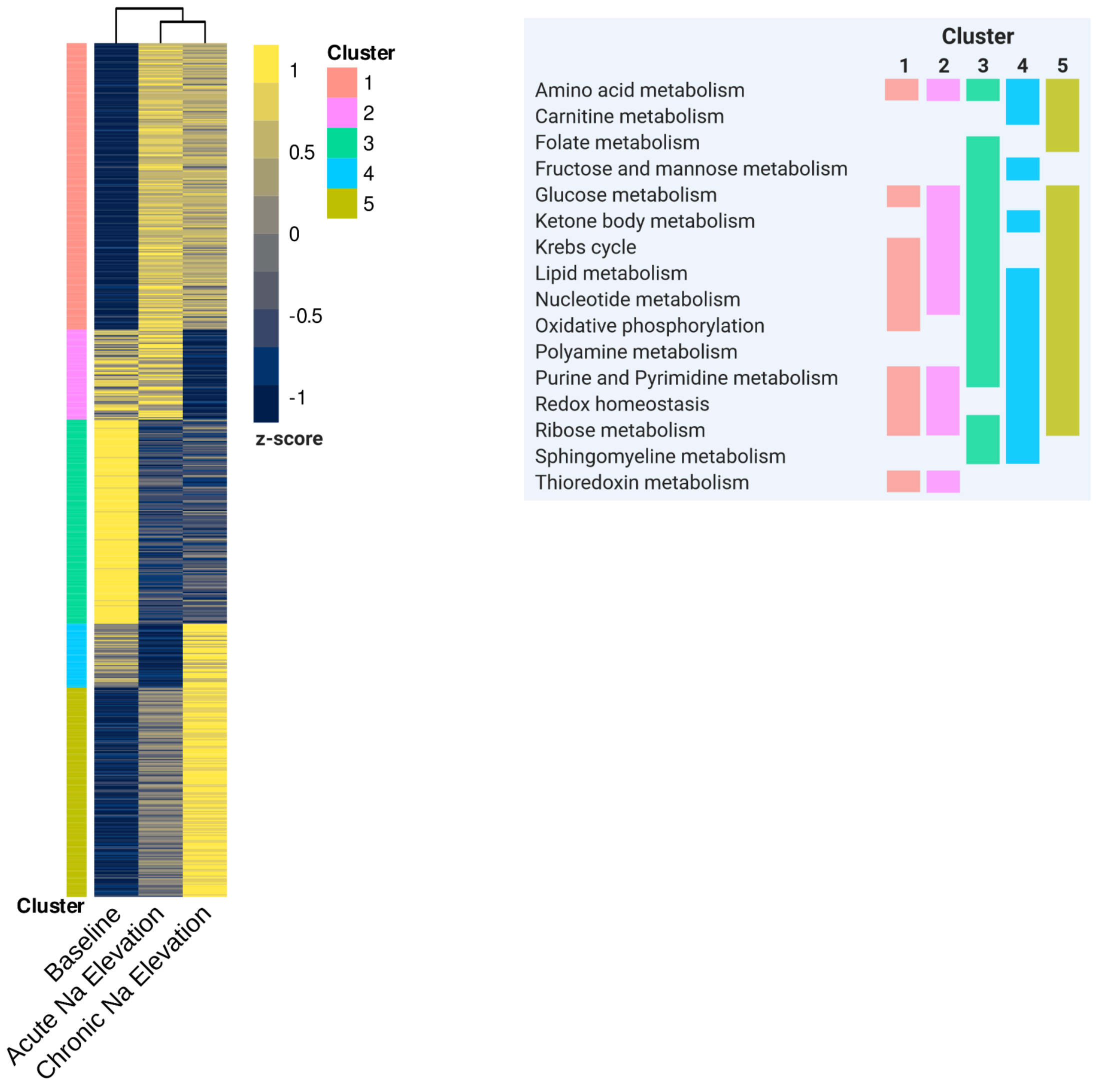

Supplementary Figure 8: Total flux rate changes in response to acute and chronic Na_i elevation.

Unsupervised hierarchical clustering of z-scored estimated flux rate changes from the indicated treatment and control groups. Five reaction clusters across 16 different metabolic pathways are annotated demonstrating metabolic adaptation in response to Na_i elevation. The table shows representation of metabolic pathways across clusters. Flux distributions were calculated by Flux balance analysis (FBA) using the mammalian network of cardiac metabolism, CardioNet. Color scale indicates the degree to which estimated flux rate changes are predicted to be respectively lower or higher in response to Na elevation. Source data are provided as a Supplementary Data 1.

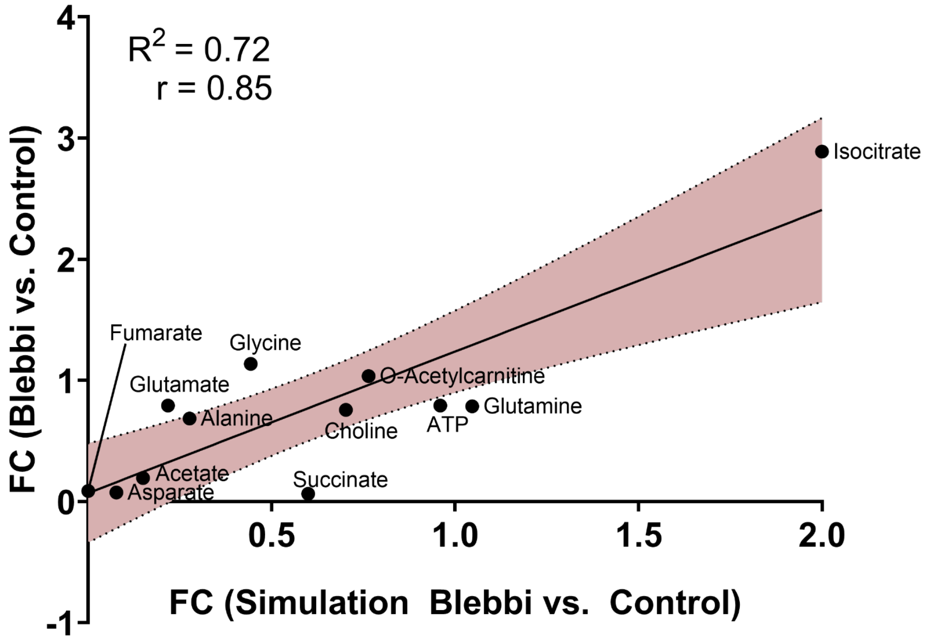

Supplementary Figure 9: Correlation between predicted and experimentally determined metabolite fractional changes (FC). Comparison between experimentally determined metabolic changes and mathematical predictions in response to acute Na_i elevation (ouabain+blebbistatin treatment). Simple linear regression analysis and Pearson correlation with α = 0.05. Graph show best-fit regression line and 95% confidence interval. (n=5 biologically independent samples). Source data are provided as a Source Data file and as a Supplementary Data 1.

Supplementary Information Table 1 Table of Primers for qRT-PCR

Gene name	PrimerBank ID	Forward Primer	Reverse Primer
Glut1	22094111a1	CAGTTCGGCTATAACACTGGTG	GCCCCCGACAGAGAAGATG
Glut4	6678015a1	GTGACTGGAACACTGGTCCTA	CCAGCCACGTTGCATTGTAG
Pdk4	7305375a1	AGGGAGGTCGAGCTGTTCTC	GGAGTGTTCACTAAGCGGTCA
Ucp3	6678495a1	CTGCACCGCCAGATGAGTTT	ATCATGGCTTGAAATCGGACC
Cpt1β	6753512a1	GCACACCAGGCAGTAGCTTT	CAGGAGTTGATTCCAGACAGGTA
36B4	6671569a1	AGATTCGGGATATGCTGTTGGC	TCGGGTCCTAGACCAGTGTTC
Mte-1	19527402a1	GTTGTGCCAACAGGATTGGAA	GCTCAGCGTCGCATTTGTC
CD36	31982474a1	ATGGGCTGTGATCGGAACTG	GTCTTCCCAATAAGCATGTCTCC
Caspase-3	12851951a1	ATGGAGAACAACAAAACCTCAGT	TTGCTCCCATGTATGGTCTTTAC
Atg3	13385890a1	ACACGGTGAAGGGAAAGGC	TGGTGGACTAAGTGATCTCCAG

Supplementary Information Table 2

	**PLM^{WT}**	**PLM^{3SA}**
Morphometric parameters		
Age (weeks)	24.2 ± 0.2	25.1 ± 0.4
Body Weight (g)	30.1 ± 0.9	29.9 ± 1.1
Wet heart weight (g)	0.3 ± 0.01	0.3 ± 0.02
Wet heart weight:tibia length (g/cm)	0.1 ± 0.01	0.1 ± 0.01
Wet heart weight:body weight (g/g) x10-	7.4 ± 0.7	7.4 ± 0.3
Plasma biochemical profile		
Adiponectin (ng/ml)	18506 ± 1371	19615 ± 1343
Adrenaline (ng/ml)	0.4 ± 0.1	0.3 ± 0.1
Alkaline transferase (IU/l)	27.8 ± 2.4	29.9 ± 3.5
Alkaline phosphatase (IU/l)	65.2 ± 6.4	65.9 ± 4.1
Creatine kinase (IU/l)	30.4 ± 6.6	28.4 ± 1.5
Lactate dehydrogenase (IU/l)	91.8 ± 17.1	89.1 ± 7.8
Free fatty acids (µmol/l)	340.6 ± 57.4	306.0 ± 36.6
High density lipoprotein (mmol/l)	1.7 ± 0.1	1.7 ± 0.1
Triacylglycerol (nmol/l)	0.8 ± 0.2	0.6 ± 0.1
Glucose (mmol/l)	10.6 ± 0.6	12.9 ± 0.5**
Insulin (µg/L)	0.1 ± 0.02	0.2 ± 0.02**
Lactate (mmol/l)	5.5 ± 0.5	5.3 ± 0.4

Table 2: PLM^{3SA} and PLM^{WT} Morphology and Biochemical Plasma Profile
Data are mean ± SEM. PLM^{WT} (n = 5-17), PLM^{3SA} (n = 7-17), independent biological samples. PLM^{WT} versus PLM^{3SA} comparison by Student's t-test (two-tailed). ** glucose P<0.008 insulin**P<0.01 versus PLM^{WT}. Source data are provided as a Source Data file.

Supplementary Information Table 3

	**Sham** (6-17)	**Banded** (6-17)
Morphological features		
Body weight (g)	26 ± 0.4	26 ± 0.3
Wet heart weight (mg)	214 ± 10	281 ± 9***
Dry heart weight (mg)	29 ± 1	53 ± 3***
Heart weight:tibia length (mg/cm)	123 ± 6	157 ± 6.1***
Heart weight:body weight (mg/g)	8.2 ± 0.40	10.66 ± 0.45***
Dry heart weight:tibia length (mg/cm)	16.4 ± 0.6	27.0 ± 1.9***
Wet lung weight:Dry lung weight (g/g)	6.9 ± 0.2	8.4 ± 0.8
In vivo function (Echocardiography)		
IVS (mm)	0.8 ± 0.02	1.2 ± 0.06***
LVID (mm)	4.1 ± 0.1	4.6 ± 0.1**
LVPW (mm)	0.7 ± 0.04	0.8 ± 0.06
LVAW (mm)	0.9 ± 0.0	1.1 ± 0.04***
MV E/A	1.8 ± 0.1	1.1 ± 0.1**
E/E’	32.6 ± 3.1	43.8 ± 6.67
FS (%)	32 ± 2.08	20 ± 1.75***
EF (%)	60 ± 3	40 ± 3***
Ex vivo function		
LVDP (mmHg)	89.2 ± 4.8	89.5 ± 15.3
Heart Rate (bpm)	283 ± 40	298 ± 18
Coronary Flow (ml/min)	3.4 ± 0.5	3.5 ± 0.6

Table 3: Morphological features, *in vivo* cardiac function Echo assessment 5 weeks post-banding versus Sham controls, *ex vivo* Langendorff function. Data mean ± SEM). Sham control vs banded comparison by t-test (two tailed).wet heart weight, dry heart weight, ***P<0.0001, ***heart weight:tibia length P<0.0005, ***heart weigh:body weight P<0.0004, ***heart weight to tibia length P<0.0008, *** IVC P<0.0001, LVID **P<0.01, LVAW *** P<0.001 MV E/A **P<0.01, FS ***P<0.0001, EF ***P<0.0001. IVS- interventricular septum, LVID- left ventricle internal diameter, LVPW- left ventricle posterior wall, LVAW- left ventricle anterior wall, MV E/A- the ratio of early (E) to late (A) diastole trans-mitral Doppler peak flow velocity, E/E’- the ratio of trans-mitral Doppler early filling velocity (E) to tissue Doppler early diastolic mitral

annular velocity (E'), FS-fractional shortening, LVDP- left ventricular developed pressure. Source data are provided as a Source Data file.

Supplementary References

1 Eykyn, T. R. *et al.* Multiple quantum filtered (23)Na NMR in the Langendorff perfused mouse heart: Ratio of triple/double quantum filtered signals correlates with [Na]i. *Journal of molecular and cellular cardiology* **86**, 95-101, doi:10.1016/j.yjmcc.2015.07.009 (2015).
2 Ellis, D. The intracellular sodium ion concentration of sheep heart Purkinje fibres and its relationship to external sodium [proceedings]. *The Journal of physiology* **266**, 74P-75P (1977).
