# Supplementary material for: Intracellular sodium elevation reprograms cardiac metabolism
Source: Nat Commun. 2020 Aug 28;11:4337. doi: 10.1038/s41467-020-18160-x (PMC7455741; doi:10.1038/s41467-020-18160-x)
Supplement: Supplementary file 3 — Description of Additional Supplementary Files [file 41467_2020_18160_MOESM3_ESM.docx]

**Description of Supplementary Files**

**File Name: Supplementary Data 1**

**Description:**CardioNet *in silico* metabolic flux changes in response to acute and chronic Na_i_elevation.
