# Supplementary material for: Intracellular sodium elevation reprograms cardiac metabolism
Source: Nat Commun. 2020 Aug 28;11:4337. doi: 10.1038/s41467-020-18160-x (PMC7455741; doi:10.1038/s41467-020-18160-x)
Supplement: Supplementary file 5 — Reporting Summary [file 41467_2020_18160_MOESM5_ESM.pdf]

nature research

Corresponding author(s): Prof Michael Shattock

Last updated by author(s): 09.06.2020

Reporting Summary

Nature Research wishes to improve the reproducibility of the work that we publish. This form provides structure for consistency and transparency in reporting. For further information on Nature Research policies, see Authors & Referees and the Editorial Policy Checklist .

Statistics

For all statistical analyses, confirm that the following items are present in the figure legend, table legend, main text, or Methods section.

n/a	Confirmed	
[ ]	[x]	The exact sample size (*n*) for each experimental group/condition, given as a discrete number and unit of measurement
[ ]	[x]	A statement on whether measurements were taken from distinct samples or whether the same sample was measured repeatedly
[ ]	[x]	The statistical test(s) used AND whether they are one- or two-sided<br>*Only common tests should be described solely by name; describe more complex techniques in the Methods section.*
[x]	[ ]	A description of all covariates tested
[ ]	[x]	A description of any assumptions or corrections, such as tests of normality and adjustment for multiple comparisons
[ ]	[x]	A full description of the statistical parameters including central tendency (e.g. means) or other basic estimates (e.g. regression coefficient) AND variation (e.g. standard deviation) or associated estimates of uncertainty (e.g. confidence intervals)
[ ]	[x]	For null hypothesis testing, the test statistic (e.g. *F*, *t*, *r*) with confidence intervals, effect sizes, degrees of freedom and *P* value noted<br>*Give P values as exact values whenever suitable.*
[x]	[ ]	For Bayesian analysis, information on the choice of priors and Markov chain Monte Carlo settings
[ ]	[x]	For hierarchical and complex designs, identification of the appropriate level for tests and full reporting of outcomes
[ ]	[x]	Estimates of effect sizes (e.g. Cohen's *d*, Pearson's *r*), indicating how they were calculated

Our web collection on statistics for biologists contains articles on many of the points above.

Software and code

Policy information about availability of computer code

Data collection	Cardiac function data-AD Instruments Chart (v 6.0), Visualsonics Vevo 770,<br>NMR spectroscopy: Bruker TopSpin (v 2.01, v3.2.6.), Chenomx NMR Profiler (Version 8.1)<br>LC/MS and GS/MS: Analyst software (version 1.4.2),<br>RT-PCR: 7900HT SDS 2.4 Applied Biosystems<br>Western Blotting: Biorad gel 800 scanner-Image Lab Software v.6.1
Data analysis	Graphpad Prism (v 8.3), Microsoft Excel (v.16.16.15), TCA Calc (v.2.07), R Statistics (version 1.2.1335)

For manuscripts utilizing custom algorithms or software that are central to the research but not yet described in published literature, software must be made available to editors/reviewers. We strongly encourage code deposition in a community repository (e.g. GitHub). See the Nature Research guidelines for submitting code & software for further information.

Data

Policy information about availability of data

All manuscripts must include a data availability statement. This statement should provide the following information, where applicable:

- Accession codes, unique identifiers, or web links for publicly available datasets
- A list of figures that have associated raw data
- A description of any restrictions on data availability

All data available upon request. A list of figures that have associated raw metabolomics data has been provided (supplementary excel source data file).
All the databases used in the study along with appropriately accessible links in the manuscript are under the 'Data availability' section as well as in this reporting summary. KEGG database: https://www.genome.jp; PrimerBank (https://pga.mgh.harvard.edu/primerbank/, Cardionet model https://www.ebi.ac.uk/biomodels/MODEL1212040000

Field-specific reporting

Please select the one below that is the best fit for your research. If you are not sure, read the appropriate sections before making your selection.

- [x] Life sciences
- [ ] Behavioural & social sciences
- [ ] Ecological, evolutionary & environmental sciences

For a reference copy of the document with all sections, see nature.com/documents/nr-reporting-summary-flat.pdf

Life sciences study design

All studies must disclose on these points even when the disclosure is negative.

Sample size	In all parts of the project we used the minimum number of mice per experiment that allowed robust, statistically and biologically significant results to be obtained.The number of animals purchase and maintained has been estimated based on the most cost-effective power calculations in the experimental models established in the Shattock laboratory. The number of mice required to find a statistically significant difference with 80% of power is 6-7 animals per group.
Data exclusions	No data were excluded from the analyses.
Replication	All analyses were done using at least 3 independent biological replicates/mice and repeated at least twice in separate experiments at different time points. All attempts at replication were successful.
Randomization	Animals and samples were randomized into experimental groups.
Blinding	Data analysis performed blind to the phenotype.

Reporting for specific materials, systems and methods

We require information from authors about some types of materials, experimental systems and methods used in many studies. Here, indicate whether each material, system or method listed is relevant to your study. If you are not sure if a list item applies to your research, read the appropriate section before selecting a response.

Materials & experimental systems

n/a	Involved in the study	
[ ]	[x]	Antibodies
[x]	[ ]	Eukaryotic cell lines
[x]	[ ]	Palaeontology
[ ]	[x]	Animals and other organisms
[x]	[ ]	Human research participants
[x]	[ ]	Clinical data

Methods

n/a	Involved in the study	
[x]	[ ]	ChIP-seq
[x]	[ ]	Flow cytometry
[x]	[ ]	MRI-based neuroimaging

Antibodies

Antibodies used	rabbit-anti-IDH3A, Abcam,ab58641 / GR3270447-3, 1.25 ug/ml anti-rabbit, GE Healthcare NA934V / 16921443; 1:5000 dilution<br>rabbit -anti-α/ß Tubulin,Cell Signaling,2148-S / 7, anti-rabbit,GE Healthcare,NA934V / 16921443; 1:2000 dilution<br>mouse-anti-PDH, Abcam,ab110333 / GR280542-5,1 ug/ml,anti-mouse,GE Healthcare,NA931V / 9761190: 1:2000 dilution
Validation	All antibodies have been validated by the manufacturers using cell treatment validation: detecting downstream events following the treatment, knockdown validation via the expression using RNAi to knock down the gene of interest, neutralization validation by functional blockage of protein activity by antibody binding, independent antibody verification: measurement of target expression is performed using two differentially raised antibodies recognizing the same protein target.

Animals and other organisms

Policy information about studies involving animals; ARRIVE guidelines recommended for reporting animal research

Laboratory animals	Male, C57/BL6 mice (Charles River JAXTM stock number 000664),6 weeks old. Transgenic phospholemman knock in mice (PLM3SA, PLMWT 6 weeks old) on C57/BL6 background (Charles River UK). Male Wisar Rats (Harlan, UK), 8 weeks old.Animals were kept under pathogen-free conditions, 12h light–dark cycle, controlled humidity (~40%), temperature (20–22°C), and fed chow and water ad libitum.
Wild animals	No wild animals were used in the study.

Field-collected samples	No field-collected samples were used in the study.
Ethics oversight	This investigation conforms to UK Home Office Guidance on the Operation of the Animals (Scientific Procedures) Act, 1986 and was approved by King's College London ethical review committee (Home Office Project Licences MJS:PF75E5F7F and P856ECBBE).

Note that full information on the approval of the study protocol must also be provided in the manuscript.
